# Defective Ribosome Biogenesis Drives Tubular Senescence and Renal Fibrosis via IRBC–p53 Activation

**DOI:** 10.3390/cells15201888

**Published:** 2026-10-16

**Authors:** Kaixin Song, Hua Su

**Affiliations:** Department of Nephrology, Union Hospital, Tongji Medical College, Huazhong University of Science and Technology, Wuhan 430022, China; kaixins@hust.edu.cn

**Keywords:** ribosome biogenesis, impaired ribosome biogenesis checkpoint, p53, cellular senescence, kidney fibrosis, DNA damage response

## Abstract

Impaired ribosome biogenesis (RiBi) triggers the impaired ribosome biogenesis checkpoint (IRBC), a nucleolar stress pathway that stabilizes p53 and induces cellular senescence. Whether RiBi dysfunction directly drives renal interstitial fibrosis (RIF) remains unknown. Here, we combined human IgA nephropathy biopsies, murine fibrosis models, and cultured proximal tubular epithelial cells treated with the RNA polymerase I inhibitor CX5461, the p53 inhibitor pifithrin-α, siRNA-mediated POLR1A knockdown, and the DNA-damaging agent etoposide to dissect the RiBi–IRBC–p53–senescence axis. We identified defective rDNA transcription in the proximal tubules of fibrotic human kidneys and in experimental fibrosis models. Pharmacological RiBi inhibition with CX5461 induced RIF prior to functional decline. Mechanistically, RiBi impairment promoted RPL5/RPL11 binding to HDM2, stabilized p53, upregulated p21, and induced G2/M arrest, which was rescued by pifithrin-α. Genetic silencing of POLR1A recapitulated IRBC activation and senescence without causing DNA damage. Time-course analysis showed only marginal γ-H2AX elevation by CX5461 versus robust induction by etoposide. Importantly, etoposide-induced DNA damage failed to enhance RPL5/RPL11-HDM2 binding, suggesting that IRBC activation is not a general consequence of the DNA damage response. Collectively, impaired RiBi drives RIF mainly through the IRBC–p53–senescence axis under our experimental conditions, providing a mechanistic framework for understanding organ toxicities associated with RNA Pol I inhibition.

## 1. Introduction

Chronic kidney disease (CKD) represents a growing global health burden, with its prevalence escalating markedly with age and projections, indicating it will become the fifth leading cause of mortality worldwide by 2040 [1,2]. The inexorable progression of CKD is pathologically defined by renal fibrosis, manifesting as glomerulosclerosis, tubular atrophy, and, ultimately, renal interstitial fibrosis (RIF)—the final common pathway of virtually all forms of progressive kidney disease [3]. Within this fibrotic cascade, renal tubular epithelial cells (RTECs) have emerged not as passive victims but as active drivers of disease progression. In response to diverse stressors, including ischemia, toxins, and metabolic perturbations, RTECs can undergo cellular senescence—an irreversible state of cell-cycle arrest accompanied by profound phenotypic alterations [4]. This senescent state in RTECs fundamentally disrupts normal renal repair and regeneration processes, creating a permissive microenvironment for the initiation and perpetuation of fibrosis [5]. Indeed, accumulating evidence from both animal models and human studies has firmly established RTEC senescence as a critical cellular event driving the transition from acute kidney injury (AKI) to CKD and accelerating the progression of established CKD [6,7]. The clinical relevance of this mechanism is underscored by preclinical studies demonstrating that therapeutic strategies targeting senescent cells—including senolytic drugs and senomorphics—can delay CKD progression and ameliorate renal fibrosis [8]. Despite significant advances in understanding the contributions of organelle dysfunction—particularly mitochondrial damage and endoplasmic reticulum stress—to RTEC senescence and fibrosis [9,10], a comprehensive understanding of the upstream biosynthetic perturbations that initiate this program remains incomplete. In particular, the role of ribosome biogenesis (RiBi), a fundamental and energetically costly cellular process, in the pathogenesis of RTEC senescence and subsequent renal fibrosis constitutes a critical knowledge gap.

Ribosomes, the universal molecular machines responsible for protein synthesis, are macromolecular complexes comprising ribosomal proteins (RPs) and ribosomal RNAs (rRNAs). RiBi, the intricate multi-step process of ribosome assembly, commences with rDNA transcription, a rate-limiting step catalyzed exclusively by RNA polymerase I (RNA Pol I) within the nucleolus [11]. Eukaryotic RiBi requires the coordinated action of transcription factors, including SL1 and UBF, to initiate RNA Pol I-driven transcription of rDNA, producing a precursor 47S rRNA that is subsequently processed into mature 5.8S, 18S, and 28S rRNAs [12]. Concurrently, 5S rRNA is independently transcribed by RNA Pol III, while RNA Pol II transcribes RP mRNAs. These components are assembled into pre-40S and pre-60S subunits, which are exported to the cytoplasm for final 80S ribosome assembly [13,14,15]. Among the pharmacological tools available to probe RiBi function, CX5461—the first RNA Pol I inhibitor to enter clinical trials—has been widely used to dissect the specific contribution of RiBi to disease mechanisms [16,17,18]. However, emerging evidence indicates that CX5461 can also induce DNA damage through off-target effects, which may independently activate p53 and confound mechanistic interpretation [19]. To address this critical issue, the present study employs orthogonal genetic and pharmacological approaches—including siRNA-mediated POLR1A knockdown and direct comparison with the DNA-damaging agent etoposide—to distinguish IRBC-mediated p53 activation from DNA damage-dependent signaling.

The impairment of RiBi under cellular stress activates a conserved surveillance mechanism known as the impaired ribosome biogenesis checkpoint (IRBC) [20,21,22]. Mechanistically, when RiBi fails, ribosomal proteins RPL5 and RPL11, together with 5S rRNA (5S-RNP), form an inhibitory complex that binds to MDM2 (the human homolog of MDM2) and inhibits its activity toward p53 [23,24,25]. This IRBC–p53 axis is a well-established mechanism leading to cell-cycle arrest and cellular senescence in response to nucleolar stress [26,27]. Given that RTEC senescence, characterized by irreversible cell-cycle arrest, is a pivotal event in renal fibrogenesis [10,28], and considering the limited direct evidence connecting RiBi to fibrosis, we hypothesized that defective RiBi in RTECs triggers the IRBC–p53 axis, leading to cellular senescence and ultimately driving renal interstitial fibrosis. In this study, we systematically tested this hypothesis using human CKD biopsies, murine models of renal fibrosis, and mechanistic cellular assays. Our findings establish a novel pathogenic mechanism linking RiBi dysfunction to tubule-derived senescence and fibrosis, thereby providing a cellular and molecular framework relevant to CKD progression.

## 2. Materials and Methods

### 2.1. Reagents and Antibodies

CX5461, pifithrin-α (PFT-α), and etoposide were obtained from Bidepharm (Shanghai, China), Aladdin (Shanghai, China), and Macklin (Shanghai, China). TGF-β was purchased from R&D Systems (Minneapolis, MN, USA). Lotus Tetragonolobus Lectin (LTL) was obtained from Vector (Newark, CA, USA). Antibodies against GAPDH (A19056; Abclonal, Woburn, MA, USA), α-SMA (A17910; Abclonal, Woburn, MA, USA), FN (15613-1-AP; ProteinTech, Rosemont, IL, USA), p53 (10442-1-AP; ProteinTech, Rosemont, IL, USA), p21 (67362-1-Ig; ProteinTech, Rosemont, IL, USA), slc12a3 (ab95302; Abcam, Cambridge, UK), Ki67 (ab16667; Abcam, Cambridge, UK), RPL5 (A1977; Abclonal, Woburn, MA, USA), RPL11 (16277-1-AP; ProteinTech, Rosemont, IL, USA), HDM2 (sc-965; Santa Cruz Biotechnology, Dallas, TX, USA); and γ-H2AX (R381558, Zenbio, Chengdu, China) were used.

### 2.2. Patient Information

Patients with a confirmed diagnosis of primary IgA nephropathy by renal biopsy at our hospital between 1 January 2020 and 1 January 2026 were considered for the source population. The diagnosis of primary IgA nephropathy was based on renal biopsy with the exclusion of secondary causes. Inclusion criteria were: (1) patients aged ≥ 18 years; (2) biopsy-confirmed primary IgA nephropathy; and (3) an available Oxford classification T-score. Patients with secondary IgA nephropathy, other coexisting glomerular diseases, acute-phase lesions, or inadequate tissue for the planned assays were excluded. Eligible patients were stratified by their T-scores. Six patients with T0 and six with T2 were randomly selected from the respective strata using a computer-generated random number sequence. Renal tissue sections from these patients were subsequently subjected to H&E staining, Masson’s trichrome staining, and FISH. This study was approved by the Ethics Committee of Union Hospital affiliated with Tongji Medical College at the Huazhong University of Science and Technology (Approval code: 0914, date of approval: 10 June 2026), and all participants provided informed consent.

### 2.3. Cell Lines and Cell Treatment

HK-2 cells were obtained from the American Type Culture Collection (ATCC, Manassas, VA, USA; CRL-2190) and were cultured in DMEM/F12 with 10% FBS at 37 °C and 5% CO_2_. At 60% confluence, cells were stimulated for 48 h as follows: the TGF-β group received 5 ng/mL TGF-β; as shown in Figure S1, CX5461 was applied at final concentrations ranging from 20 to 160 nM, while all other experiments used a final concentration of 100 nM; the PFT-α groups at the final concentration of 20 µM; the etoposide groups at the final concentration of 5 µM; and the control groups received a matching volume of DMSO. Cells were harvested after the 48 h treatment period.

### 2.4. siRNA Transfection

HK-2 cells were transfected with siRNA targeting POLR1A (siPOLR1A) or a negative control siRNA (NC) using RNAiBoost transfection reagent (Yeasen, China) according to the manufacturer’s protocol. Briefly, cells were seeded in 6-well plates and transfected at about 50% confluence. siRNA transfection reagent complexes were prepared and added to the cells. Cells were harvested 48 h post-transfection for subsequent analyses. siRNA sequences are provided in Supplementary Table S1.

### 2.5. Animals

The UUO model was induced in 8-week-old male C57BL/6 mice housed under a standard 12 h light/dark cycle with free access to food and water. Under anesthesia with 1% sodium pentobarbital, a midline laparotomy was performed. The left ureter was isolated and ligated at two locations with a 5-0 suture. After returning the organs to the abdominal cavity, the incision was closed in layers, and the wound was disinfected with povidone-iodine.

A mouse model of RiBi impairment was established using 8-week-old male C57BL/6 mice also housed under standard conditions. Based on body weight and experimental grouping, the mice received either a vehicle control (normal saline) or CX5461 at doses ranging from 0.6 to 1.0 mg/kg body weight via alternate-day intraperitoneal injection for a duration of 3 weeks. All animal experiments performed in this study were approved by the animal medicine committee of Tongji Medical College at the Huazhong University of Science and Technology (Approval code: HUST-IACUC-2026-0069, date of approval: 2 January 2026).

### 2.6. Immunofluorescence Staining

Following PBS washes, cells were fixed (4% PFA), permeabilized (0.5% Triton X-100), and blocked (3% BSA). They were then incubated with anti-Ki67 (Abcam, ab16667; 1:200) at 4 °C overnight, followed by a fluorescent secondary antibody for 1 h at room temperature in the dark. After counterstaining nuclei with DAPI-containing mounting medium, images were acquired using a fluorescence microscope (488 nm excitation). Signal intensity was analyzed via ImageJ (version 1.54r).

### 2.7. Nascent RNA Staining

Cells were incubated with 500 μM EU for 2 h (37 °C, 5% CO_2_) before being fixed (4% PFA) and quenched with glycine. After permeabilization (0.5% Triton X-100), EU incorporation was detected using a commercial Apollo staining kit per the manufacturer’s protocol (30 min incubation in the dark). Post-staining washes (Triton X-100, methanol, PBS) were performed before mounting in DAPI-containing medium. Imaging and mean fluorescence intensity quantification were carried out using a fluorescence microscope and ImageJ, respectively. EU incorporation reflects overall nascent RNA synthesis and is not specific to RNA Pol I or RiBi. Since the majority of nascent RNA consists of rRNA, this indicator was used as a supplementary detection tool.

### 2.8. Multiplex Immunofluorescence Staining

Mice were injected with EU (5 mg/mouse) 6 h before tissue harvest. Deparaffinized and antigen-retrieved tissue sections were permeabilized (0.5% Triton X-100) and blocked (5% BSA). Sequential staining was then performed, first with anti-slcl2a3 primary (1:300, 4 °C overnight) and the corresponding secondary antibody (1:300, room temperature, 1 h), followed by EU detection via a commercial Apollo staining kit (room temperature, 30 min, dark) and then finally with LTL (1:100, room temperature, 3 h). After counterstaining with DAPI, images were captured by confocal microscopy (488/567/647 nm) and analyzed for mean fluorescence intensity using ImageJ.

### 2.9. CCK8

Cell proliferation was determined using a CCK-8 assay. Briefly, 5,000 cells were seeded per well in a 96-well plate, and their viability was measured at days 0, 1, 2, and 3. For each measurement, 10 μL of enhanced CCK-8 reagent was added to each well, and after 4 h of incubation at 37 °C, the absorbance was read at 450 nm and 620 nm using a microplate reader. The proliferation capacity was calculated from the dual-wavelength data.

### 2.10. Flow Cytometry

For cell-cycle analysis, cells were harvested, washed with PBS, and fixed overnight in 70% ethanol at 4 °C. After washing, cells were resuspended in a propidium iodide (PI) staining solution (prepared as per the manufacturer’s protocol) and incubated at 37 °C for 30 min in the dark. Samples were then analyzed on a flow cytometer (excitation: 488 nm), and the data were processed using FlowJo (version 10.10) software to assess DNA content and light scatter properties.

### 2.11. SA-β-Gal Staining

Cells were washed with PBS, fixed for 15 min at room temperature, and washed again. Subsequently, cells were incubated overnight at 37 °C with a freshly prepared SA-β-Gal staining working solution (made as per the manufacturer’s protocol). Senescent cells were identified by the presence of blue staining under a standard light microscope.

### 2.12. Hematoxylin and Eosin (H&E) Staining

Deparaffinized and rehydrated kidney sections were subjected to standard H&E staining. Briefly, sections were stained with hematoxylin, differentiated (1% acid alcohol), blued (1% ammonia water), and dehydrated through a graded ethanol series. After counterstaining with eosin, sections were cleared in xylene and mounted with neutral balsam. Images were acquired using a light microscope.

### 2.13. Masson’s Trichrome Staining

Deparaffinized and rehydrated sections were stained following Masson’s trichrome protocol as standard. Briefly, nuclei were stained with an iron hematoxylin mixture (10 min), followed by differentiation and bluing. Cytoplasm and muscle fibers were stained with Lichun Red (5 min), and the collagen was specifically stained with Aniline Blue (2 min) after treatment with acetic acid and phosphomolybdic acid. Sections were then dehydrated, cleared in xylene, and mounted. Collagen-positive areas (blue) were quantified from microscope images using ImageJ software (version 1.54r).

### 2.14. Co-Immunoprecipitation (Co-IP)

Cells were lysed on ice and centrifuged. A portion of the supernatant was saved as the input after boiling with a loading buffer. The remainder was incubated overnight at 4 °C with 2 μg of a HDM2 antibody or control IgG. Protein A/G Agarose beads were then added for 3 h of incubation at 4 °C. After washing the beads with cold PBS, bound proteins were eluted in 2× loading buffer by boiling. Input, IgG control, and IP samples were analyzed by Western blotting.

### 2.15. Fluorescence In Situ Hybridization (FISH)

After deparaffinization and rehydration, tissue sections underwent microwave-assisted antigen retrieval in pure water. Sections were then digested with Proteinase K (37 °C, 3 min), washed, and pre-hybridized. They were subsequently hybridized overnight at 37 °C with a target probe diluted in hybridization buffer. Following hybridization, sections were stringently washed with graded SSC buffers (42 °C), PBS, and ethanol. After drying, sections were mounted with DAPI-containing anti-fade medium and imaged using a confocal microscope. Signal quantification (mean fluorescence intensity and positive cell percentage) was performed with ImageJ software. FISH probes were designed and synthesized by RiboBio Co., Ltd (Guangzhou, China).

### 2.16. Total Protein Extraction

Proteins were extracted from tissues or cells using a RIPA-based lysis buffer supplemented with PMSF, protease, and phosphatase inhibitors. Mice tissues were homogenized, and all samples were sonicated. After centrifugation (12,000× *g*, 30 min, 4 °C), the protein concentration of the supernatant was quantified by BCA assay. Proteins were then denatured by boiling in 5× loading buffer and stored at −20 °C.

### 2.17. Western Blotting

Proteins (20 μg per lane) were separated by SDS-PAGE using hand-cast gels and transferred to a PVDF membrane. After blocking with 5% skim milk, membranes were probed with specific primary antibodies against GAPDH (Proteintech, Proteintech, Rosemont, IL, USA, 60004-1-Ig, 1:1000), α-SMA (Proteintech, 14395-1-AP, 1:1000), FN (Proteintech, 15613-1-AP, 1:1000), p53 (Proteintech, 60283-2-Ig, 1:1000), and p21 (CST, CST, Danvers, Massachusetts, SAD, #2947, 1:1000) at 4 °C overnight. Following incubation with an HRP-conjugated secondary antibody, signals were detected with an ECL reagent and quantified using ImageJ software.

### 2.18. Total RNA Extraction

Total RNA was isolated from tissues or cells using a commercial reagent. Following lysis (with homogenization for tissues), chloroform was added, and the mixture was centrifuged. The aqueous phase was collected, mixed with isopropanol, and incubated at −20 °C to precipitate RNA. The RNA pellet was obtained by centrifugation, washed with 75% ethanol, and dissolved in DEPC-water. RNA concentration and the A260/A280 ratio were determined using a NanoDrop 2000 spectrophotometer (Thermo Fisher Scientific, Waltham, MA, USA).

### 2.19. Reverse Transcription

cDNA was synthesized from 5 μg total RNA using the ABScript III Reverse Transcriptase kit according to the manufacturer’s protocol. Briefly, RNA was incubated with gDNA Remover Mix and RT Mix in a 100 μL reaction volume. The thermal cycling conditions were: 37 °C for 2 min, 55 °C for 15 min, and 85 °C for 5 min, with a final hold at 4 °C.

### 2.20. Quantitative Real-Time PCR (RT-qPCR)

The RT-qPCR reaction was performed using a 20 μL reaction mixture containing SYBR Green Premix, specific forward and reverse primers, cDNA template, and nuclease-free water. The thermal cycling protocol consisted of UDG incubation (37 °C, 2 min), initial denaturation (95 °C, 3 min), followed by 40 cycles of 95 °C for 5 s and 60 °C for 30 s. Gene expression was quantified using the 2(−ΔΔCt) method. Primer sequences are provided in Supplementary Table S2.

### 2.21. Quantification and Statistical Analysis

Data were analyzed and visualized with GraphPad Prism (version 10.4.1). For histological quantification, a minimum of five random fields per tissue sample were analyzed, and their mean value represented the sample. Two group comparisons were made by unpaired *t*-test, and multi-group comparisons were made by one-way ANOVA followed by Bonferroni’s post hoc test, with statistical significance set at *p* < 0.05—ns, not significant; * *p* < 0.05; ** *p* < 0.01; *** *p* < 0.001; and **** *p* < 0.0001.

## 3. Results

### 3.1. RiBi Was Suppressed in Renal Tissues from Patients with CKD

**Figure 1:**
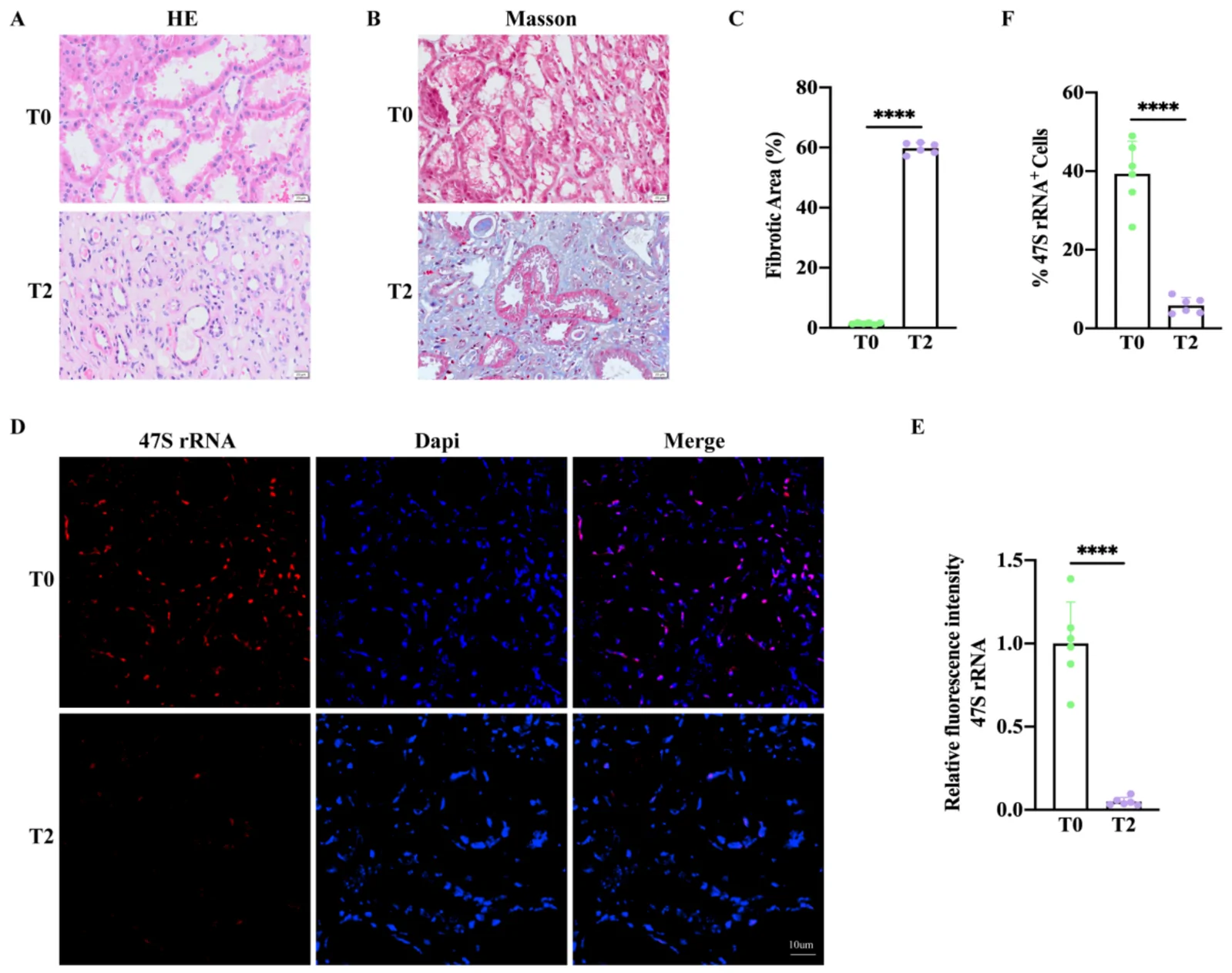
RiBi was suppressed in renal tissues from patients with CKD. (**A**) Representative HE staining of renal tissues from patients with Oxford classification T0 and T2 scores. (**B**,**C**) Representative Masson’s trichrome staining of renal tissues and quantitative analysis of fibrosis-positive areas in T0 and T2 groups. (**D**–**F**) Representative FISH images and analysis of 47S rRNA expression detected by FISH in T0 and T2 renal tissues. *n* = 6, unpaired *t*-tests were used. ns, not significant; **** *p* < 0.0001.

To investigate the clinical relevance of RiBi in renal fibrosis, we assessed RiBi status in kidney tissues from patients with IgA nephropathy, stratified by the Oxford classification into T0 (minimal fibrosis) and T2 (severe fibrosis) groups. HE staining revealed advanced chronic damage in T2 tissues, characterized by tubular atrophy, interstitial inflammation, and the loss of normal tubular architecture (Figure 1A). Masson’s trichrome staining confirmed extensive collagen deposition in the T2 group, establishing severe RIF (Figure 1B,C). To directly probe RiBi activity, we measured 47S pre-rRNA levels by FISH. T2 tissues exhibited marked downregulation of 47S rRNA, reflected by both a reduced mean fluorescence intensity and a lower proportion of positive cells compared to T0 tissues (Figure 1D–F). These findings provide clinical correlational evidence linking impaired RiBi to human RIF.

### 3.2. RiBi Was Selectively Suppressed in Proximal Tubules During Renal Fibrosis

**Figure 2:**
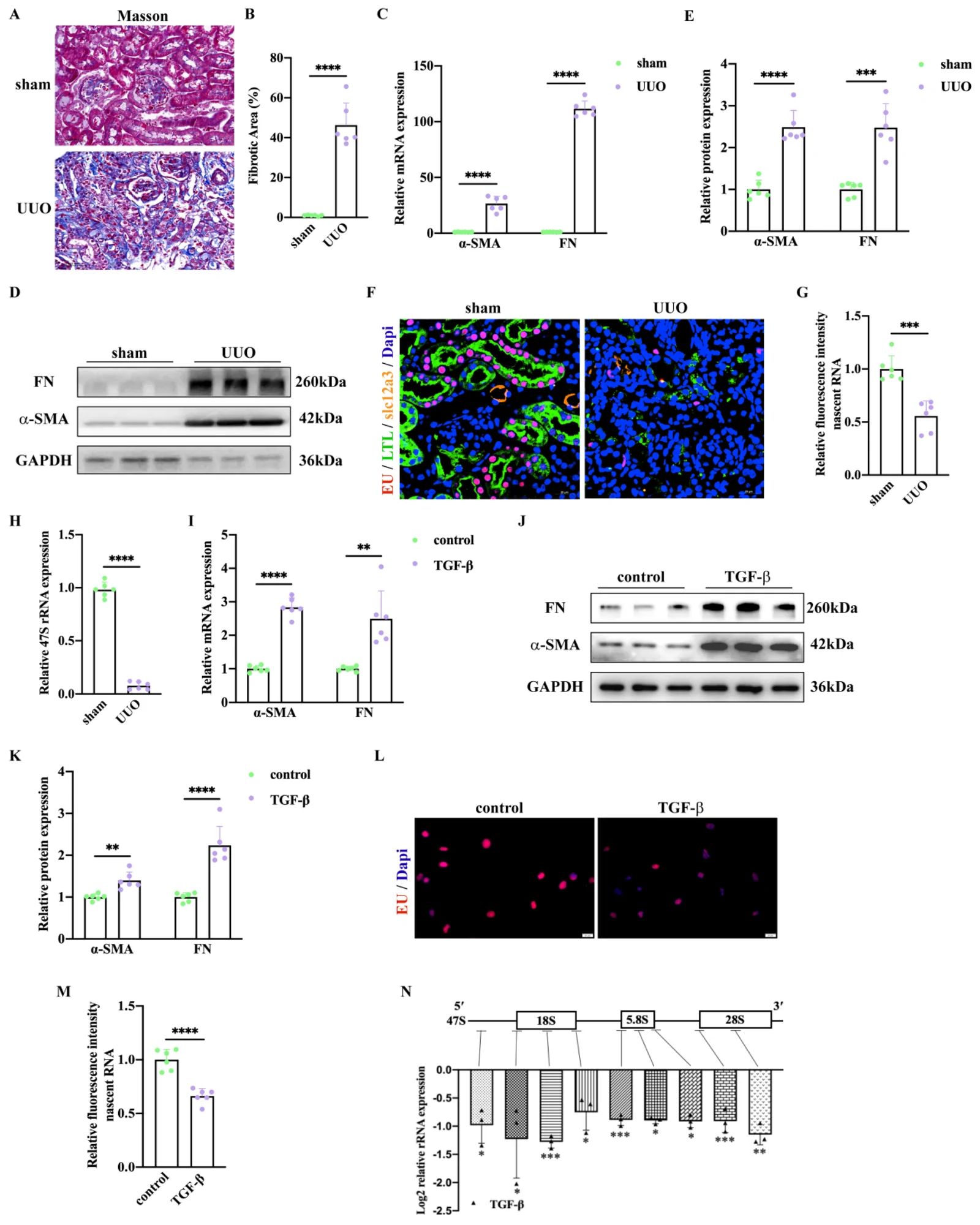
RiBi was selectively suppressed in proximal tubules during renal fibrosis in UUO mice and TGF-β-stimulated HK-2 cells. (**A**,**B**) Representative Masson’s trichrome staining and quantitative analysis of the fibrosis-positive areas in sham and UUO mice. (**C**) mRNA expression levels of α-SMA and FN in renal tissues from sham and UUO groups, as determined by RT-qPCR. (**D**,**E**) Protein expression levels of α-SMA and FN, as detected by Western blotting, and their corresponding semi-quantitative analyses. (**F**) Representative images of multiplex immunofluorescence staining for nascent RNA synthesis (labeled by EU, red) in kidney tissues, while proximal tubules and distal tubules were marked by LTL (green) and slc12a3 (orange). (**G**) Quantitative analysis of the mean fluorescence intensity of EU staining. (**H**) Expression level of 47S rRNA, as measured by RT-qPCR. (**I**) mRNA expression levels of α-SMA and FN in control and TGF-β-treated HK-2 cells, as determined by RT-qPCR. (**J**,**K**) Protein expression levels of α-SMA and FN, as detected by Western blotting, and their corresponding semi-quantitative analyses. (**L**,**M**) Nascent RNA synthesis was assessed by EU staining and quantified by mean fluorescence intensity analysis. (**N**) Expression levels of 47S, 18S, 5.8S, and 28S rRNAs in control and TGF-β-treated cells were measured by RT-qPCR using primers specific to different sites from the 5′ to 3′ ends. n = 6 (N, n = 3), unpaired *t*-tests were used. ns, not significant; ** *p* < 0.01; *** *p* < 0.001; and **** *p* < 0.0001.

We next employed complementary in vivo and in vitro models to determine the spatial dynamics of RiBi during renal fibrosis. In the unilateral ureteral obstruction (UUO) mouse model, successful induction of fibrosis was confirmed by Masson’s trichrome staining, which revealed exacerbated collagen deposition in UUO kidneys (Figure 2A,B), and by the elevated expression of fibrosis markers α-SMA and FN at both the mRNA and protein levels (Figure 2C–E). To evaluate RiBi activity in situ, we performed multiplex immunofluorescence staining using LTL to identify proximal tubules, slc12a3 to mark distal tubules, and EU labeling to detect nascent RNA synthesis. Notably, EU incorporation was substantially decreased specifically in the proximal tubules of UUO mice (Figure 2F,G). Consistent with this finding, RT-qPCR analysis revealed significantly reduced 47S rRNA expression in UUO kidneys (Figure 2H).

To determine whether this suppression could be recapitulated in a defined in vitro system, we treated HK-2 cells with TGF-β, the master pro-fibrotic cytokine. TGF-β stimulation successfully induced a fibrotic response, as evidenced by increased α-SMA and FN expression (Figure 2I–K). Under these pro-fibrotic conditions, nascent RNA synthesis was significantly reduced (Figure 2L,M). We further quantified rRNA levels using primers spanning from the 5′ to 3′ ends of the primary transcript [29]. Both the primary 47S rRNA transcript and its processed products (18S, 5.8S, and 28S rRNAs) were markedly decreased (Figure 2N). Collectively, these data provide consistent evidence that RiBi is selectively and comprehensively suppressed in proximal tubular epithelial cells during renal fibrosis.

### 3.3. Pharmacological Inhibition of rDNA Transcription Directly Induced RIF in Mice

**Figure 3:**
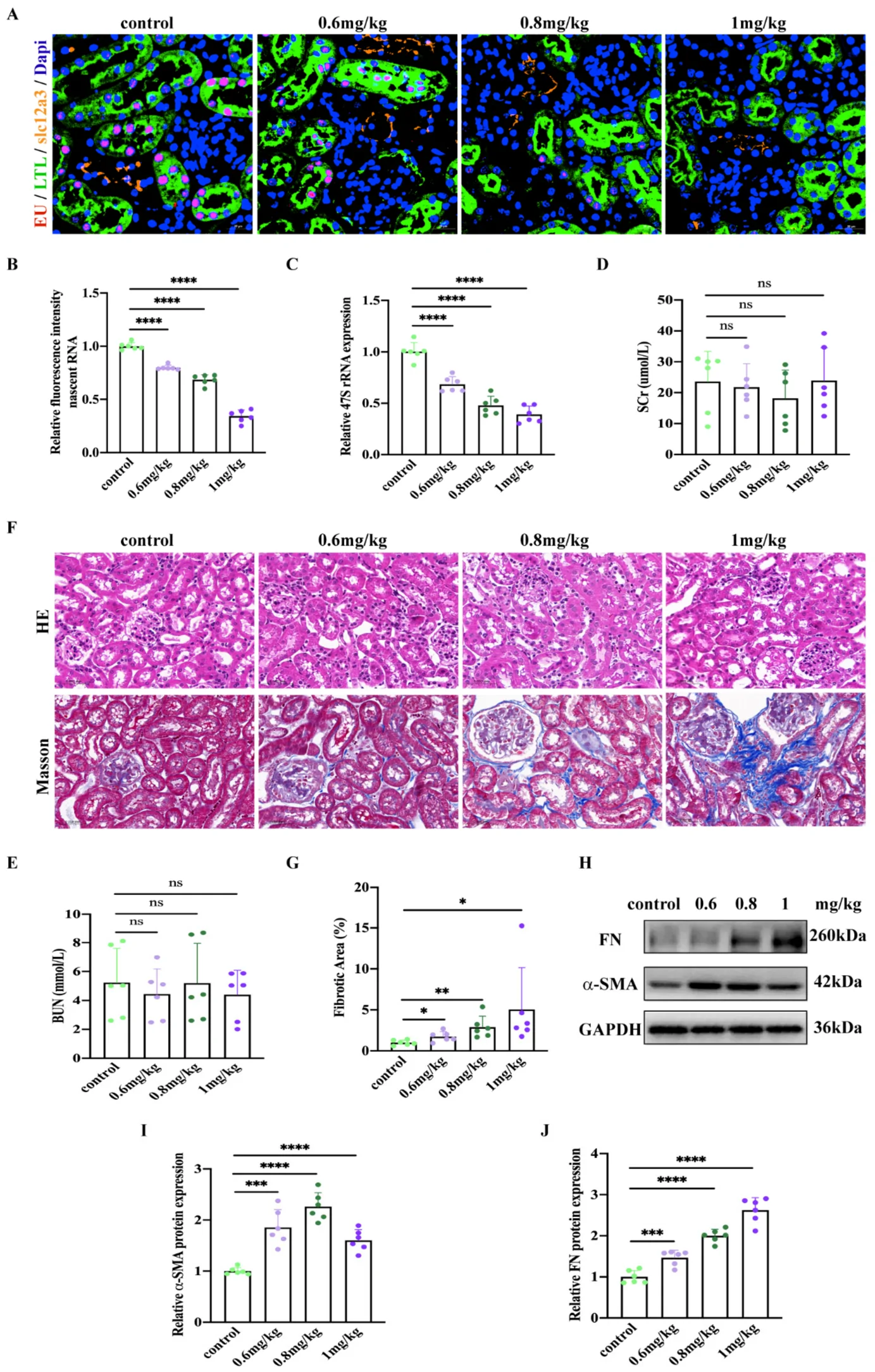
Inhibition of rDNA transcription inducing RiBi impairment led to RIF in mice. (**A**,**B**) Nascent RNA synthesis in kidney tissues from control and CX5461-treated mice was detected by multiplex immunofluorescence staining and quantified by mean fluorescence intensity analysis of EU labeling. (**C**) Expression level of 47S rRNA in renal tissues was measured by RT-qPCR. (**D**,**E**) Serum creatinine and blood urea nitrogen levels in control and CX5461-treated mice. (**F**) Representative images of H&E and Masson’s trichrome staining of kidney sections. (**G**) Quantitative analysis of the fibrosis-positive areas from Masson’s trichrome staining. (**H**–**J**) Protein expression levels of α-SMA and FN in renal tissues were detected by Western blotting and semi-quantitatively analyses. n = 6, one-way ANOVA followed by Bonferroni’s post hoc test were used. ns, not significant; * *p* < 0.05; ** *p* < 0.01; *** *p* < 0.001; and **** *p* < 0.0001.

To establish a causal relationship between RiBi impairment and renal fibrosis, we treated mice with CX5461, an RNA polymerase I inhibitor that suppresses rDNA transcription. Dose-dependent suppression of RiBi was achieved, as demonstrated by reduced nascent RNA synthesis and 47S rRNA levels in kidney tissues (Figure 3A–C). Strikingly, this RiBi inhibition induced significant renal pathology without altering standard renal function markers (serum creatinine and BUN; Figure 3D,E), positioning the RiBi defect as an early driver of kidney fibrosis that precedes functional decline. Most importantly, CX5461-treated kidneys developed clear histopathological fibrosis. H&E and Masson’s staining revealed escalating tubular damage and collagen deposition (Figure 3F,G), which was corroborated by dose-dependent increases in α-SMA and FN protein expression (Figure 3H–J). These data reflected that impairing RiBi via rDNA transcription inhibition was sufficient to trigger the onset and progression of RIF.

### 3.4. Direct RiBi Inhibition Induced a Pro-fibrotic Phenotype in HK-2 Cells

To determine whether RiBi suppression autonomously triggers fibrotic activation in RTECs, we applied CX5461 to HK-2 cells. As shown in Supplementary Figure S1, this treatment resulted in the concentration-dependent suppression of RiBi, as evidenced by decreased nascent RNA synthesis and 47S rRNA levels (Figure S1A–C). Concomitantly, the protein expression of α-SMA and FN was significantly elevated in a dose-dependent manner (Figure S1D–F). These results demonstrate that RiBi dysfunction directly and autonomously triggered a pro-fibrotic response in RTECs, corroborating our in vivo observations.

### 3.5. RiBi Suppression Activated the IRBC and Induced RTEC Senescence

**Figure 4:**
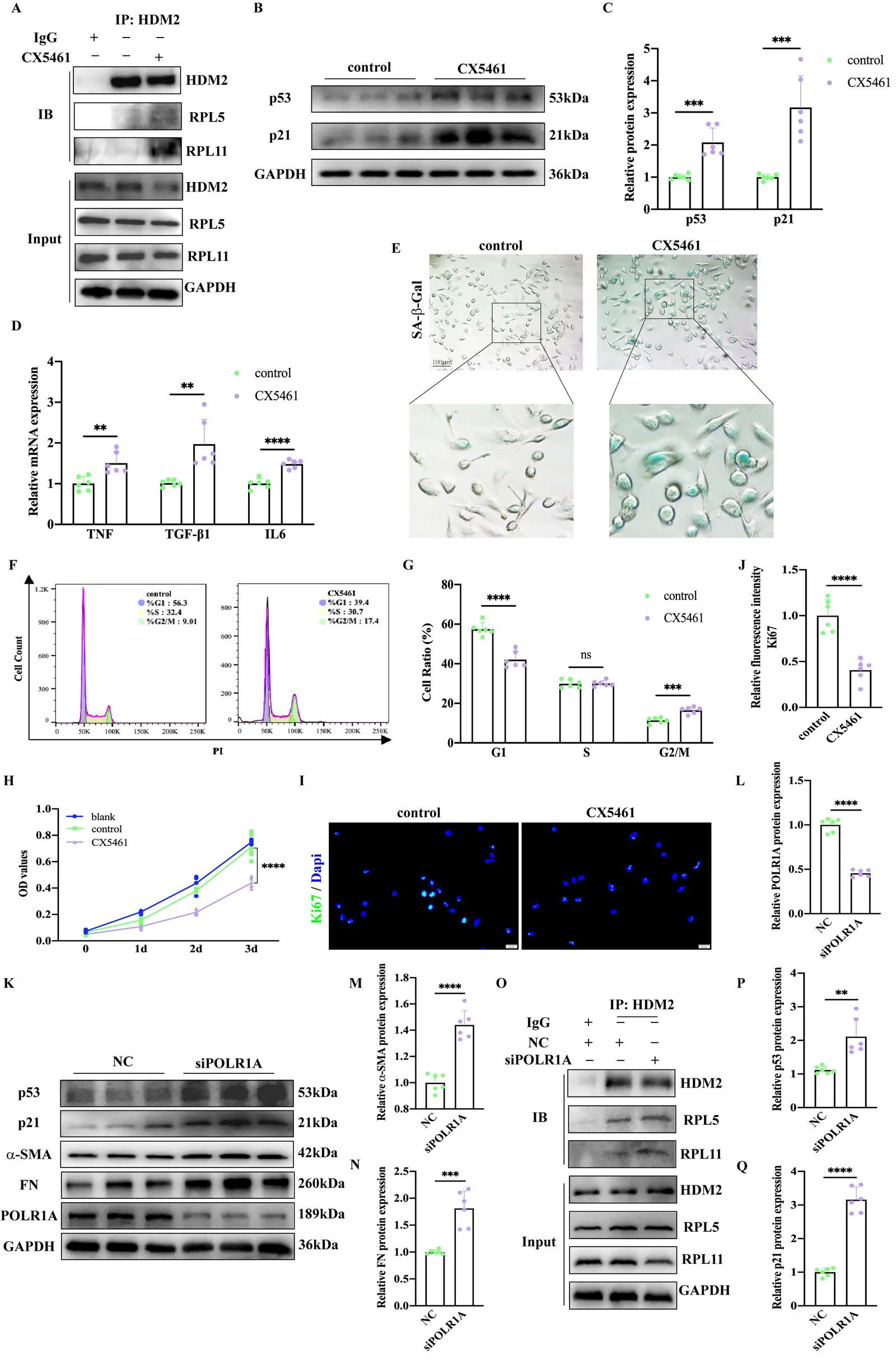
The suppression of RiBi activated the IRBC, leading to senescence in RTECs. (**A**) Interactions between HDM2 and ribosomal proteins RPL5/RPL11 in control and CX5461-stimulated HK-2 cells were assessed by co-immunoprecipitation (Co-IP). (**B**,**C**) Protein expression levels of p53 and p21 were detected by Western blotting and semi-quantitatively analyzed. (**D**) Expression levels of TNF, TGF-β1, and IL6 mRNAs in control and CX5461-treated cells were measured by RT-qPCR. (**E**) SA-β-gal activity was measured by staining in control and CX5461-treated cells. (**F**,**G**) Cell-cycle distribution was analyzed by flow cytometry, and percentages of cells in each phase were statistically summarized. (**H**) Cell proliferation was evaluated using the CCK-8 assay. (**I**,**J**) Expression of Ki67 was detected by immunofluorescence and quantified by mean fluorescence intensity analysis. (**K**–**N**,**P**,**Q**) Protein expression levels of POLR1A, p53, p21, α-SMA, and FN were detected by Western blotting and semi-quantitatively analyzed. (**O**) Interactions between HDM2 and ribosomal proteins RPL5/RPL11 in NC and siPOLR1A-treated cells were assessed by Co-IP. n = 6, unpaired *t*-tests were used in (**C**,**D**,**G**,**J**,**L**–**N**,**P**,**Q**) and one-way ANOVA followed by Bonferroni’s post hoc test in **H**. ns, not significant ; ** *p* < 0.01; *** *p* < 0.001; and **** *p* < 0.0001.

We next dissected the molecular mechanism by which RiBi suppression leads to fibrosis. In HK-2 cells, CX5461-induced RiBi impairment triggered IRBC activation, as evidenced by the enhanced binding of RPL5 and RPL11 to HDM2 (Figure 4A; Figure S3A,B) and subsequent p53 protein stabilization (Figure 4B,C). The stabilized p53 initiated a robust senescent program, as marked by p21 upregulation (Figure 4B,C), and induced senescence-related secretory phenotypes (SASP) (Figure 4D) with increased SA-β-Gal activity (Figure 4E), as well as a definitive G2/M phase cell-cycle arrest (Figure 4F,G). Consequently, cell proliferation was significantly inhibited, as confirmed by CCK-8 assay and Ki67 immunofluorescence staining (Figure 4H–J).

Furthermore, we constructed a RiBi model by downregulating POLR1A expression. Among three siPOLR1A constructs, we selected the one with the highest downregulation efficiency for subsequent experiments (Figure S2A). The results demonstrated that POLR1A downregulation significantly inhibited nascent RNA synthesis and 47S rRNA expression (Figure S2B,C), leading to a marked increase in fibrosis-related markers (Figure 4K–N). Mechanistically, the suppression of POLR1A expression also activated the IRBC (Figure 4O, Figure S3C,D), elevated the expression levels of p53 and p21 (Figure 4K,P,Q), enhanced SASP and SA-β-gal activities (Figure S2E,F), induced G2/M phase cell-cycle arrest (Figure S2G,H), and suppressed cell proliferation (Figure S2I–K). These data suggest a pathogenic axis: RiBi impairment activates the IRBC, which drives p53-dependent tubular cell senescence, ultimately contributing to renal fibrosis.

### 3.6. p53 Inhibition Attenuated RiBi Impairment and Induced Senescence and Fibrosis

**Figure 5:**
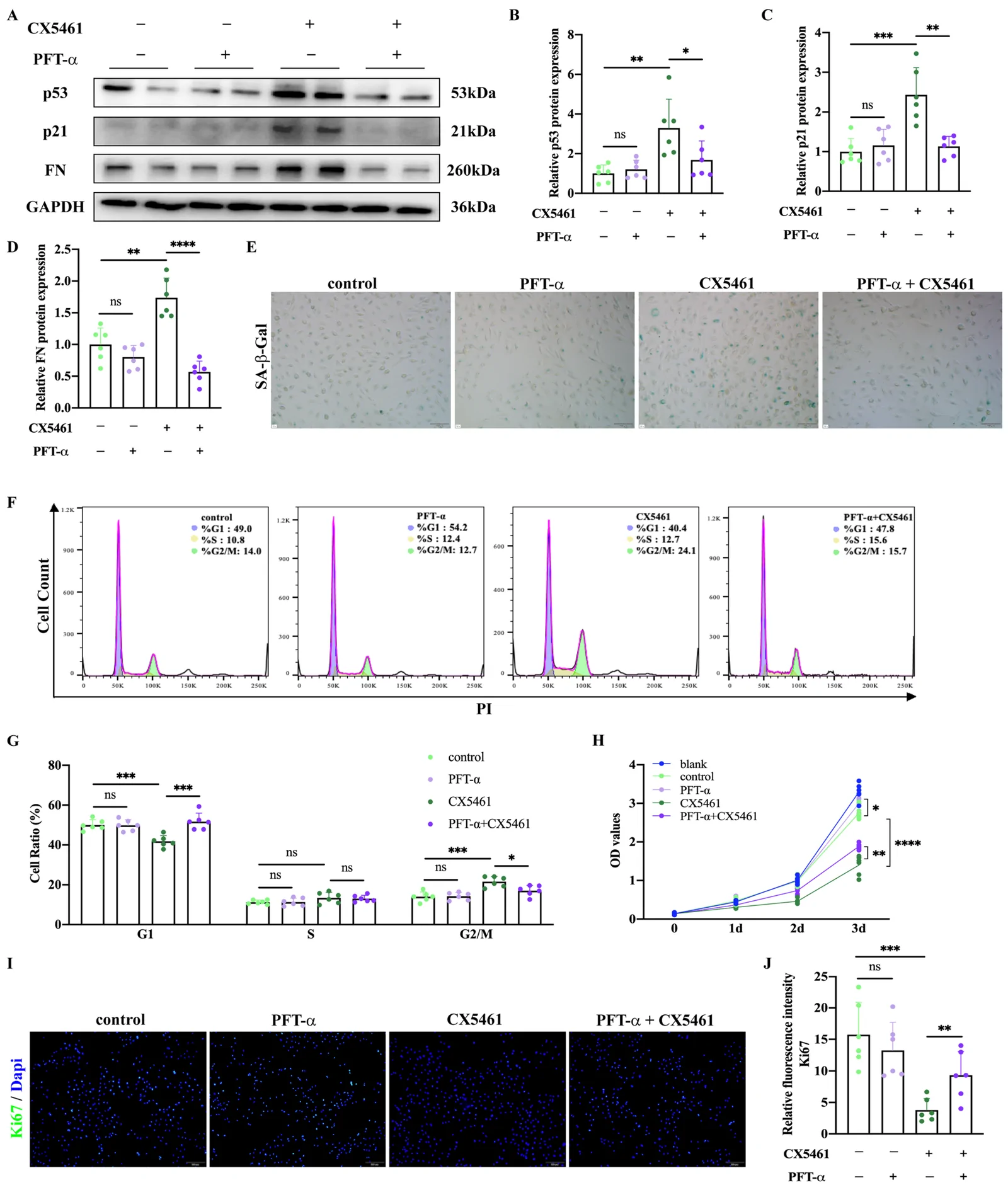
Inhibition of p53 alleviates cell senescence and fibrosis induced by RiBi impairment. (**A**–**D**) Protein expression levels of p53, p21, and FN were detected by Western blotting and semi-quantitatively analysis in control, PFT-α, CX5461, and PFT-α+CX5461 groups. (**E**) SA-β-gal activity was measured by staining. (**F**,**G**) Cell-cycle distribution was analyzed by flow cytometry, and percentages of cells in each phase were statistically summarized. (**H**) Cell proliferation was evaluated using the CCK-8 assay. (**I**,**J**) Expression of Ki67 was detected by immunofluorescence and quantified by mean fluorescence intensity analysis. n = 6, one-way ANOVA followed by Bonferroni’s post hoc test were used. ns, not significant; * *p* < 0.05; ** *p* < 0.01; *** *p* < 0.001; and **** *p* < 0.0001.

To validate p53 as the critical downstream effector of IRBC activation, we treated HK-2 cells with the p53 inhibitor pifithrin-α (PFT-α) in the presence of CX5461. Western blot analysis confirmed that PFT-α significantly suppressed p53 protein levels. Consequently, the CX5461-induced upregulation of the senescence marker p21 and the fibrotic marker FN was markedly alleviated (Figure 5A–D). Consistent with this, SA-β-gal activity was significantly reduced by PFT-α and CX5461 co-treatment (Figure 5E). Flow cytometry analysis further revealed that PFT-α substantially rescued the G2/M phase cell-cycle arrest triggered by RiBi impairment (Figure 5F,G). Moreover, functional assays using CCK-8 and Ki67 immunofluorescence staining demonstrated that the suppression of cell proliferation caused by CX5461 was significantly reversed upon p53 inhibition (Figure 5H–J). Notably, PFT-α treatment alone had no appreciable effect on these parameters, supporting a role for p53 as a downstream mediator of the IRBC–senescence axis following RiBi dysfunction. Furthermore, in another model of RiBi dysfunction induced by POLR1A inhibition, we also obtained consistent results, wherein PTF-α also alleviated cellular senescence and the fibrotic phenotypes induced by siPOLR1A (Figure S4).

### 3.7. RiBi Impairment Primarily Activates the IRBC–p53 Axis

**Figure 6:**
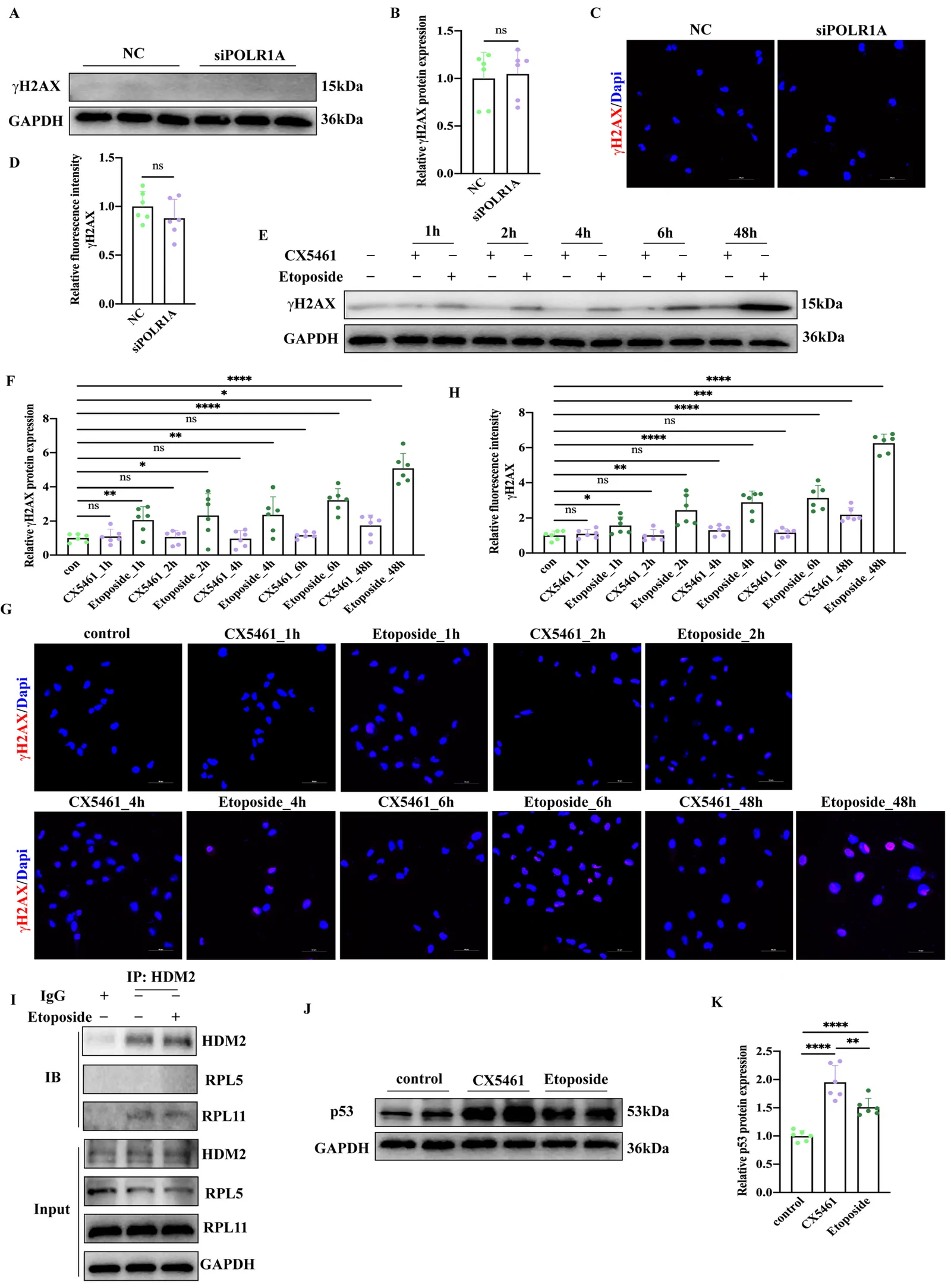
RiBi impairment primarily activates the IRBC–p53 axis. (**A**,**B**) Protein expression levels of γ-H2AX were detected by Western blotting and semi-quantitatively analysis of HK-2 cells transfected with siNC or siPOLR1A. (**C**,**D**) Expression of γ-H2AX was detected by immunofluorescence and quantified by mean fluorescence intensity analysis in cells transfected with siNC or siPOLR1A. (**E**,**F**) Protein expression levels of γ-H2AX were detected by Western blotting and semi-quantitatively analysis of HK-2 cells treated by CX5461 or etoposide at different time points. (**G**,**H**) Expression of γ-H2AX was detected by immunofluorescence and quantified by mean fluorescence intensity analysis. (**I**) Interactions between HDM2 and ribosomal proteins RPL5/RPL11 in control and etoposide-treated cells were assessed by Co-IP. (**J**,**K**) Protein expression levels of p53 were detected by Western blotting and semi-quantitatively analysis of HK-2 cells treated by CX5461 or etoposide. n = 6, unpaired *t*-tests were used in B and D; one-way ANOVA followed by Bonferroni’s post hoc test were used in (**F**,**H**,**K**). ns, not significant; * *p* < 0.05; ** *p* < 0.01; *** *p* < 0.001; and **** *p* < 0.0001.

To determine whether RiBi inhibition activates the DNA damage response (DDR), we first assessed γ-H2AX levels following siRNA-mediated POLR1A knockdown. Neither Western blotting nor immunofluorescence analysis revealed any increase in γ-H2AX in siPOLR1A-transfected cells compared to NCs (Figure 6A–D), indicating that pure RiBi impairment does not induce detectable DNA double-strand breaks. We next compared the DDR activation kinetics between CX5461 and etoposide, a classic topoisomerase II inhibitor causing double-strand breaks in DNA. Etoposide induced a robust and sustained increase in γ-H2AX from 1 to 48 h, with levels reaching 4-8-fold above controls according to Western blotting and immunofluorescence (Figure 6E–H). In striking contrast, CX5461 treatment caused only a marginal increase in γ-H2AX at 48 h (~2-fold), with no detectable elevation at earlier time points (Figure 6E–H). This near-absence of γ-H2AX signal indicates that CX5461, at the concentration used in this study, does not trigger substantial DNA double-strand breaks. Furthermore, the expression levels of 53BP1 also exhibited similar results. Inhibition of POLR1A did not lead to increased 53BP1 expression. Etoposide induced a peak expression of 53BP1 at 4–6 h, followed by a decline at 48 h. In contrast, CX5461 elicited only a faint fluorescent signal of 53BP1 expression at 6 h, but with no statistically significant difference (Figure S5). To further distinguish between IRBC-mediated and DDR-mediated p53 stabilization, we examined IRBC complex formation. CX5461 markedly enhanced the binding of RPL5 and RPL11 to HDM2, whereas etoposide did not (Figure 6I, Figure S3E,F), suggesting that DNA damage does not activate the IRBC under our experimental conditions. Consistent with their distinct mechanisms, CX5461 induced a greater accumulation of total p53 protein than etoposide at 48 h (Figure 6J,K), reflecting the efficiency of IRBC-mediated inhibition of MDM2-dependent p53 degradation over DDR-mediated p53 stabilization. Collectively, these data suggest that RiBi impairment stabilizes p53 primarily through IRBC activation rather than DDR.

## 4. Discussion

The present study identifies impaired RiBi as a novel and direct pathogenic driver of RIF. By integrating evidence from human CKD biopsies, experimental murine models, and mechanistic cellular assays, we delineate a pathological axis wherein RiBi dysfunction triggers the IRBC, stabilizes p53, and culminates in RTEC senescence. This work not only establishes RiBi as an early event in fibrogenesis but also provides a mechanistic framework for understanding the organ toxicities associated with RNA polymerase I inhibitors, thereby bridging fundamental cell biology with clinical pharmacology.

Our initial observation of suppressed RiBi, manifested by reduced 47S rRNA levels, in fibrotic human CKD kidneys was consistently replicated in both animal and cellular models of renal fibrosis. Multiplex immunofluorescence analysis further revealed that this suppression occurred selectively in proximal tubular epithelial cells, identifying a specific cellular compartment vulnerable to RiBi dysfunction. The causal relationship between RiBi impairment and fibrosis was established using CX5461, an RNA polymerase I inhibitor that directly induces RIF in mice. Critically, RiBi inhibition induced fibrosis and preceded a detectable decline in renal function, mirroring the clinical scenario in CKD where pathological remodeling often antedates measurable functional impairment [30]. This temporal dissociation suggests that RiBi dysfunction may represent an early, actionable target for intervention before irreversible renal damage occurs.

The pathogenic role of RiBi impairment in fibrosis is consistent with independent lines of evidence. Pluripotent stem-cell-derived megakaryocytes exhibiting pro-fibrotic phenotypes concurrently display suppressed RiBi [31], while mutations in PARN—a gene essential for ribosomal subunit maturation—have been linked to idiopathic pulmonary fibrosis [32]. These observations support the notion that RiBi dysfunction may represent a conserved mechanism underlying fibrosis across different organs and disease contexts. This broad pathogenic role is further substantiated by recent studies implicating RiBi loss in cardiac aging, which was rescued by RiBi-promoting interventions, such as LAV-BPIFB4 gene therapy [33].

Mechanistically, our study delineated a complete signaling cascade linking RiBi impairment to renal fibrosis through IRBC activation and tubular senescence. We demonstrated that RiBi inhibition in RTECs triggered the assembly of the IRBC complex, as evidenced by enhanced HDM2-RPL5/RPL11 binding and subsequent p53 stabilization. This aligns with the established role of the MDM2–p53 signaling axis in multi-organ fibrosis [34,35,36,37] and supports p53 as the key downstream effector of RiBi dysfunction [38,39]. The stabilized p53 executed a canonical senescence program characterized by p21 upregulation, induction of a senescence-associated secretory phenotype (SASP), increased SA-β-gal activity, and a prominent G2/M phase cell-cycle arrest. This G2/M arrest is particularly relevant to renal fibrosis, as sustained G2/M-arrested tubular cells have been shown to produce pro-fibrotic cytokines that drive fibroblast activation and extracellular matrix deposition [40]. In the in vivo RiBi inhibition model, we also detected a significant increase in p53 and p21 levels (Supplementary Figure S6). The functional necessity of this axis was confirmed by p53 inhibition with PFT-α, which rescued the senescent phenotype and attenuated the fibrotic response, supporting p53 as a principal downstream effector. However, we acknowledge that PFT-α may have off-target effects, and future genetic validation using TP53 knockdown would further strengthen this conclusion.

A potential concern with the use of CX5461 is its reported capacity to induce DNA damage independently of RNA Pol I inhibition, which could confound the interpretation of p53-dependent phenotypes. Our study systematically addresses this concern through three complementary approaches. First, a time-course analysis of γ-H2AX revealed that CX5461 induces only marginal DNA double-strand breaks (~2-fold at 48 h) compared with the robust and sustained damage elicited by etoposide (>2-fold from 1 h onward, reaching 4–8-fold by 48 h). Furthermore, in contrast to etoposide, which significantly elevated 53BP1 levels, CX5461 induced an increase of less than 1.5-fold only at 6 h, and this effect was not statistically significant. These indicated that DDR activation by CX5461 is negligible under our experimental conditions. Second, co-immunoprecipitation analysis demonstrated that etoposide-induced DNA damage does not enhance RPL5/RPL11-HDM2 binding, suggesting that IRBC activation is not a general consequence of the DDR under our experimental conditions. Third, and most importantly, genetic silencing of POLR1A—which directly inhibits RiBi without causing DNA damage—recapitulated IRBC activation, p53 stabilization, and downstream senescence phenotypes. The qualitative concordance between genetic and pharmacological RiBi inhibition suggests that RiBi impairment per se, rather than off-target DDR activation, is a primary driver of the IRBC–p53–senescence–fibrosis axis under our experimental conditions.

Our findings, together with recent independent reports, reveal a consistent cell type specific pattern of Pol I inhibition in fibrotic diseases. A previous study by Pang et al. demonstrated that CX5461 at 1 μM suppresses cardiac fibroblast activation via p53-dependent mechanisms, but they concluded that this effect was independent of RiBi inhibition based on the absence of pre-rRNA changes at a single time point [38]. Intriguingly, comet assays in their study detected no significant DNA strand breaks, even at this 10-fold higher concentration, consistent with our observation that CX5461 does not induce substantial DNA damage. More recently, Luo et al. reported that Pol I expression and RiBi activity are elevated in activated hepatic stellate cells and in mouse models of liver fibrosis, and that pharmacological inhibition of Pol I with CX5461 suppresses HSC activation and attenuates liver fibrosis progression [41]. Together, these studies suggest a potential cell-type-specific pattern: in mesenchymal-lineage cells including cardiac fibroblasts and HSCs, RiBi may support the activated, proliferative phenotype that drives fibrosis, and its inhibition appears anti-fibrotic. In contrast, as we demonstrate here, in renal tubular epithelial cells—a parenchymal epithelial lineage—RiBi is essential for cellular homeostasis, and its impairment triggers IRBC–p53-mediated senescence, leading to a SASP-driven paracrine pro-fibrotic response. Our data thus provide a mechanistic framework for understanding the potential renal toxicities of Pol I inhibitors and highlight the need for cell-type-specific delivery strategies to maximize therapeutic efficacy while minimizing adverse effects.

Our findings also have important clinical implications regarding the use of CX5461 and related RNA Pol I inhibitors. Phase I clinical trials of CX5461 in patients with hematologic malignancies and solid tumors have reported renal dysfunction and hepatotoxicity as notable adverse events [42,43]. Our study provides a potential mechanistic explanation for the renal toxicities observed with CX5461, suggesting that RiBi inhibition in renal tubular epithelium may trigger IRBC–p53-mediated senescence and fibrosis. The hepatic injury and systemic inflammatory changes observed in our study (Supplementary Figure S7) further underscore the potential for systemic adverse effects during RNA Pol I inhibition. This raises the possibility that monitoring markers of RiBi dysfunction or senescence could serve as early indicators of organ toxicity during CX5461 therapy. However, whether IRBC activation is the universal mechanism underlying these pro-fibrotic signatures in extra-renal organs warrants dedicated organ-specific investigation with histological validation.

Intriguingly, we observed a marked increase in peripheral immune cell counts following CX5461 treatment (Supplementary Figure S7C–I), which contrasts with the immunosuppressive effects reported in systemic lupus erythematosus models [44]. This discrepancy suggests that the immunological consequences of RiBi inhibition may be context dependent and potentially influenced by the underlying disease state, the specific immune microenvironment, or the duration of treatment. The role of immune dysregulation in RiBi-mediated fibrosis merits further investigation.

Several limitations of this study should be acknowledged. First, our mechanistic investigation focused primarily on the initiation step of RiBi (rDNA transcription), leaving the contribution of subsequent RiBi steps, such as rRNA processing, ribosomal subunit assembly, and nuclear export, to fibrosis unresolved. Second, our pathological analysis centered on RTECs, whereas the consequences of RiBi disruption in other renal cell types (e.g., podocytes, mesangial cells, endothelial cells, and immune cells) remain unexplored. Third, while we observed a pro-fibrotic effect of RiBi impairment in kidney, establishing IRBC activation as a universal mechanism requires dedicated organ-specific investigation. Fourth, the lack of tubule-specific RiBi-deficient genetic models limits our ability to definitively establish cell-autonomous effects in vivo, and the RPL5/RPL11 knockdown experiments would strengthen our conclusion. Fifth, our patient sample size was limited, and larger, independent validation cohorts are needed to confirm the clinical association between RiBi suppression and RIF progression. Sixth, future studies employing nucleolin or fibrillarin immunofluorescence would complement functional nucleolar activity data. Finally, it remains possible that severe fibrosis secondarily impairs RiBi, creating a feed-forward loop that amplifies fibrogenesis, and thus warrants further exploration.

## 5. Conclusions

In conclusion, our findings suggest that impaired ribosome biogenesis is a pathogenic driver of renal interstitial fibrosis operating through the IRBC–p53–senescence axis. This mechanism not only advances our understanding of renal fibrogenesis but also provides a conceptual foundation for developing diagnostic and therapeutic strategies targeting the RiBi-IRBC pathway in chronic kidney disease. Given the emerging clinical use of RNA Pol I inhibitors, our findings also have direct implications for understanding and monitoring potential renal toxicities associated with these agents.

## Data Availability

The data that support the findings of this study are available upon request from the corresponding author.

## Supplementary Materials

The following supporting information can be downloaded at: https://www.mdpi.com/article/10.3390/cells15201888/s1. Figure S1: Inhibition of RiBi upregulated fibrotic markers in vitro. Figure S2: The impairment of RiBi caused by downregulation of POLR1A led to cellular senescence. Figure S3: IRBC-level analysis. Figure S4: Inhibition of p53 alleviates cell senescence and fibrosis induced by RiBi impairment. Figure S5: 53BP1 foci levels in RiBi deficiency and DNA damage. Figure S6: Senescence markers of the in vivo RiBi impairment model. Figure S7: CX5461-induced RiBi impairment led to hepatotoxicity and altered hematological parameters in mice. Table S1: Sequences for siRNA. Table S2: Primer sequences for RT-qPCR.

## Abbreviations

The following abbreviations are used in this manuscript:

CKD	Chronic Kidney Disease
RiBi	Ribosome Biogenesis
DDR	DNA Damage Response
RIF	Renal Interstitial Fibrosis
RP	Ribosomal Protein
RTEC	Renal Tubular Epithelial Cell
SA-β-gal	Senescence-Associated β-Galactosidase
SASP	Senescence-Associated Secretory Phenotype
TGF-β	Transforming Growth Factor-β
UUO	Unilateral Ureteral Obstruction
PFT-α	Pifithrin-α
EU	5-Ethynyluridine
IRBC	Impaired Ribosome Biogenesis Checkpoint

## References

[1] Levey A.S., Inker L.A., Coresh J. (2015). Chronic Kidney Disease in Older People. JAMA.

[2] Xie Y., Bowe B., Mokdad A.H., Xian H., Yan Y., Li T., Maddukuri G., Tsai C.-Y., Floyd T., Al-Aly Z. (2018). Analysis of the Global Burden of Disease study highlights the global, regional, and national trends of chronic kidney disease epidemiology from 1990 to 2016. Kidney Int..

[3] Nath K.A. (1992). Tubulointerstitial changes as a major determinant in the progression of renal damage. Am. J. Kidney Dis..

[4] Huang R., Fu P., Ma L. (2023). Kidney fibrosis: From mechanisms to therapeutic medicines. Signal Transduct. Target. Ther..

[5] Chen J., Zhang H., Yi X., Dou Q., Yang X., He Y., Chen J., Chen K. (2024). Cellular senescence of renal tubular epithelial cells in acute kidney injury. Cell Death Discov..

[6] Castellano G., Franzin R., Sallustio F., Stasi A., Banelli B., Romani M., De Palma G., Lucarelli G., Divella C., Battaglia M. (2019). Complement component C5a induces aberrant epigenetic modifications in renal tubular epithelial cells accelerating senescence by Wnt4/βcatenin signaling after ischemia/reperfusion injury. Aging.

[7] Funk N.D., Sinning J., Schaser P., Soerensen-Zender I., Wulfmeyer V.C., Schmidt-Ott K., Brand K., Halle S., Schmitt R. (2025). Mechanisms of natural killer cell-mediated clearance of senescent renal tubular epithelial cells. Front. Cell Dev. Biol..

[8] Cai W., Zhang H., Li Z., Cai M., Yu S., Chen P., Song X. (2025). Tissue Fibrosis Decoded via Cellular Senescence: Mechanisms, Treatments, and Emerging Technologies. Aging Dis..

[9] Yu F., Meng Y., Wang X., Chen J., He J., Yi X., He Y., Chen K. (2025). Protein lactylation of citrate synthase promotes the AKI-CKD transition by activating the NLRP3 inflammasome. Cell Rep..

[10] Huang L., Shen Y., Pan X., Li J., Li C., Ruan L., He S., Huang L., Liu K., Zhao X. (2025). Noncanonical function of Pannexin1 promotes cellular senescence and renal fibrosis post-acute kidney injury. Nat. Commun..

[11] Drygin D., Rice W.G., Grummt I. (2010). The RNA polymerase I transcription machinery: An emerging target for the treatment of cancer. Annu. Rev. Pharmacol. Toxicol..

[12] Pilsl M., Engel C. (2020). Structural basis of RNA polymerase I pre-initiation complex formation and promoter melting. Nat. Commun..

[13] Jiao L., Liu Y., Yu X.Y., Pan X., Zhang Y., Tu J., Song Y.-H., Li Y. (2023). Ribosome biogenesis in disease: New players and therapeutic targets. Signal Transduct. Target. Ther..

[14] Hurt E., Cheng J., Baβler J., Iwasa J., Beckmann R. (2023). SnapShot: Eukaryotic ribosome biogenesis I. Cell.

[15] Hurt E., Iwasa J., Beckmann R. (2024). SnapShot: Eukaryotic ribosome biogenesis II. Cell.

[16] Ferret L., Pol J.G., Sauvat A., Stoll G., Alvarez-Valadez K., Muller A., Le Naour J., Peyre F., Anagnostopoulos G., Martins I. (2025). Lysosomal membrane permeabilization enhances the anticancer effects of POLR1 (RNA polymerase I) transcription inhibitors. Autophagy.

[17] Ferreira R., Schneekloth J.S., Panov K.I., Hannan K.M., Hannan R.D. (2020). Targeting the RNA Polymerase I Transcription for Cancer Therapy Comes of Age. Cells.

[18] Li H.X., He Y.M., Fei J., Guo M., Zeng C., Yan P.J., Xu Y., Qin G., Teng F.-Y. (2025). The G-quadruplex ligand CX-5461: An innovative candidate for disease treatment. J. Transl. Med..

[19] Trojnar J., Cottini M.V., Dudek M., Monnereau C., Khrouz L., Hsueh K.-W., Liu Y.-C., Huang P.-Y., Jamroskovic J., Deiana M. (2026). G-quadruplex DNA reprograms the photochemistry of CX-5461 toward radical-driven anticancer activity. Nucleic Acids Res..

[20] Payea M.J., Anerillas C., Tharakan R., Gorospe M. (2021). Translational Control during Cellular Senescence. Mol. Cell. Biol..

[21] Nishimura K., Kumazawa T., Kuroda T., Katagiri N., Tsuchiya M., Goto N., Furumai R., Murayama A., Yanagisawa J., Kimura K. (2015). Perturbation of ribosome biogenesis drives cells into senescence through 5S RNP-mediated p53 activation. Cell Rep..

[22] Lindström M.S., Bartek J., Maya-Mendoza A. (2022). P53 at the crossroad of DNA replication and ribosome biogenesis stress pathways. Cell Death Differ..

[23] Dönig J., Mende H., Davila Gallesio J., Wagner K., Hotz P., Schunck K., Piller T., Hölper S., Uhan S., Kaulich M. (2023). Characterization of nucleolar SUMO isopeptidases unveils a general p53-independent checkpoint of impaired ribosome biogenesis. Nat. Commun..

[24] Pelletier J., Thomas G., Volarević S. (2018). Ribosome biogenesis in cancer: New players and therapeutic avenues. Nat. Rev. Cancer.

[25] Hannan K.M., Soo P., Wong M.S., Lee J.K., Hein N., Poh P., Wysoke K.D., Williams T.D., Montellese C., Smith L.K. (2022). Nuclear stabilization of p53 requires a functional nucleolar surveillance pathway. Cell Rep..

[26] Aguilar-Garrido P., Velasco-Estévez M., Navarro-Aguadero M., Otero-Sobrino Á., Ibáñez-Navarro M., Marugal M., Hernández-Sánchez M., Malaney P., Rodriguez A., Benitez O. (2025). The tumor suppressor HNRNPK induces p53-dependent nucleolar stress to drive ribosomopathies. J. Clin. Investig..

[27] Moudry P., Chroma K., Bursac S., Volarevic S., Bartek J. (2022). RNA-interference screen for p53 regulators unveils a role of WDR75 in ribosome biogenesis. Cell Death Differ..

[28] Giuliani K.T.K., Nag P., Adams B.C., Wang X., Hong S., Grivei A., Johnston R.L., Waddell N., Ho K.K.C., Tian Y. (2025). Human proximal tubular epithelial cell interleukin-1 receptor signalling triggers G2/M arrest and cellular senescence during hypoxic kidney injury. Cell Death Dis..

[29] Espinoza J.A., Zisi A., Kanellis D.C., Carreras-Puigvert J., Henriksson M., Hühn D., Watanabe K., Helleday T., Lindström M.S., Bartek J. (2020). The antimalarial drug amodiaquine stabilizes p53 through ribosome biogenesis stress, independently of its autophagy-inhibitory activity. Cell Death Differ..

[30] Reiss A.B., Jacob B., Zubair A., Srivastava A., Johnson M., De Leon J. (2024). Fibrosis in Chronic Kidney Disease: Pathophysiology and Therapeutic Targets. J. Clin. Med..

[31] Flosdorf N., Böhnke J., de Toledo M.A.S., Lutterbach N., Lerma V.G., Graßhoff M., Olschok K., Gupta S., Tharmapalan V., Schmitz S. (2024). Proinflammatory phenotype of iPS cell-derived JAK2 V617F megakaryocytes induces fibrosis in 3D in vitro bone marrow niche. Stem Cell Rep..

[32] Montellese C., Montel-Lehry N., Henras A.K., Kutay U., Gleizes P.E., O’Donohue M.F. (2017). Poly(A)-specific ribonuclease is a nuclear ribosome biogenesis factor involved in human 18S rRNA maturation. Nucleic Acids Res..

[33] Cattaneo M., Beltrami A.P., Thomas A.C., Spinetti G., Alvino V.V., Avolio E., Veneziano C., Rolle I.G., Sponga S., Sangalli E. (2023). The longevity-associated BPIFB4 gene supports cardiac function and vascularization in ageing cardiomyopathy. Cardiovasc. Res..

[34] Li M., Wang Y., Hu Z., Huang S., Chen P., Chen L., Wu J., Wu Z., Yao S., Yang Y. (2025). PTEN-mediated senescence of lung epithelial cells drives ventilator-induced pulmonary fibrosis. Theranostics.

[35] Lu Y., Ji T., Ye Z., Yan J., Wang C., Chen J., Jin Z., Zhu Y., Cai X., Wang Y. (2025). Gelsolin’s Protective Role in MASH through F-Actin Regulation and P53 Degradation. Adv. Sci..

[36] Su H., Ye C., Lei C.T., Tang H., Zeng J.Y., Yi F., Zhang C. (2020). Subcellular trafficking of tubular MDM2 implicates in acute kidney injury to chronic kidney disease transition during multiple low-dose cisplatin exposure. FASEB J..

[37] Burke R.M., Lighthouse J.K., Quijada P., Dirkx R.A., Rosenberg A., Moravec C.S., Alexis J.D., Small E.M. (2018). Small proline-rich protein 2B drives stress-dependent p53 degradation and fibroblast proliferation in heart failure. Proc. Natl. Acad. Sci. USA.

[38] Pang S., Chen Y., Dai C., Liu T., Zhang W., Wang J., Cui X., Guo X., Jiang F. (2021). Anti-fibrotic effects of p53 activation induced by RNA polymerase I inhibitor in primary cardiac fibroblasts. Eur. J. Pharmacol..

[39] Cho C.J., Nguyen T., Rougeau A.K., Huang Y.Z., To S., Lin X., Gamage S.T., Meier J.L., Mills J.C. (2025). Inhibition of Ribosome Biogenesis In Vivo Causes p53-Dependent Death and p53-Independent Dysfunction. Cell. Mol. Gastroenterol. Hepatol..

[40] Canaud G., Brooks C.R., Kishi S., Taguchi K., Nishimura K., Magassa S., Scott A., Hsiao L.-L., Ichimura T., Terzi F. (2019). Cyclin G1 and TASCC regulate kidney epithelial cell G(2)-M arrest and fibrotic maladaptive repair. Sci. Transl. Med..

[41] Luo W., Yi L., Zhang Y., Zhou J., Li S., Abouelnazar F.A., Wang Y., Yan Y. (2025). Targeting RNA Polymerase I Inhibits Ribosome Biogenesis to Block Liver Fibrosis Progression. Liver Int..

[42] Khot A., Brajanovski N., Cameron D.P., Hein N., Maclachlan K.H., Sanij E., Lim J., Soong J., Link E., Blombery P. (2019). First-in-Human RNA Polymerase I Transcription Inhibitor CX-5461 in Patients with Advanced Hematologic Cancers: Results of a Phase I Dose-Escalation Study. Cancer Discov..

[43] Hilton J., Gelmon K., Bedard P.L., Tu D., Xu H., Tinker A.V., Goodwin R., Laurie S.A., Jonker D., Hansen A.R. (2022). Results of the phase I CCTG IND.231 trial of CX-5461 in patients with advanced solid tumors enriched for DNA-repair deficiencies. Nat. Commun..

[44] Wu Y., Tsai H.I., Zhu H., Zhang Y., Liu S., Guo P., Zhang Z., Zhang Z., Wen X., Wang D. (2024). CX-5461 ameliorates disease in lupus-prone mice by triggering B-cell ferroptosis via p53-SLC7A11-ALOX12 pathway. Free Radic. Biol. Med..

