# Supplementary material for: Defective Ribosome Biogenesis Drives Tubular Senescence and Renal Fibrosis via IRBC–p53 Activation
Source: Cells. 2026 Oct 16;15(20):1888. doi: 10.3390/cells15201888 (PMC13702928; doi:10.3390/cells15201888)
Supplement: Supplementary file 1 [file cells-15-01888-s001.zip › cells-4537807 Table S1,2.pdf]

Table S1. Sequences for siRNA

Gene	Forward	Reverse
siPOLR1A_1	AGCGAGAUGGACAAACUAA	UUAGUUUGUCCAUCUCGCU
siPOLR1A_2	GCCCAGACAUGUACAUCAA	UUGAUGUACAUGUCUGGGC
siPOLR1A_3	GGGGACAUUCUGCUACUGA	UCAGUAGCAGAAUGUCCCC

Table S2. Primer sequences for RT-qPCR

Gene	Forward	Reverse
GAPDH_Mouse	AATGGTGAAGGTCGGTGT	GTGGAGTCATACTGGAACATGTAG
α-SMA_Mouse	TGGCACCACTCTTTCTATAACG	GGTCATTTTCTCCCGGTTGG
FN_Mouse	ACAGTCCAGCAAGCAGCAAGC	TGGTGGTCACTCTGTAGCCTGTC
47S rRNA_Mouse	TTTTTGGGGAGGTGGAGAGTC	CTGATACGGGCAGACACAGAAC
GAPDH_Human	ACCAAATCCGTTGACTCCGAC	CTCCTGTTCGACAGTCAGCC
α-SMA_Human	CTCTGGACGCACAACTGGCATC	GGCATGGGGCAAGGCATAGC
FN_Human	AATAGATGCAACGATCAGGACA	GCAGGTTTCCTCGATTATCCTT
18S rRNA_Human	GATGGTAGTCGCCGTGCC	GCCTGCTGCCTTCCTTGG
5.8S rRNA_Human	ACTCGGCTCGTGCGTC	GCGACGCTCAGACAGG
28S rRNA_Human	GTGACGCGCATGAATGGA	TGTGGTTTCGCTGGATAGTAGGT
47S_5′ rRNA_Human	GAACGGTGGTGTGTCGTT	GCGTCTCGTCTCGTCTCACT
18S_5′ rRNA_Human	GCCGCGCTCTACCTTACCTACCT	CAGACATGCATGGCTTAATCTTTG
18S_3′ rRNA_Human	AGTCGTAACAAGGTTTCCGTAGGT	CCTCCGGGCTCCGTTAAT
5.8S_5′ rRNA_Human	TACGACTCTTAGCGGTGGATCA	TCACATTAATTCTCGCAGCTAGCT
5.8S_3′ rRNA_Human	GAATTGCAGGACACATTGATCATC	GGCAAGCGACGCTCAGA
28S_5′ rRNA_Human	CCGAGACGCGACCTCAGAT	TCCGCTGACTAATATGCTTAAATTCA
TNF_Human	CTCCTTCCTGATCGTGGCAG	ATGGGCTACAGGCTTGTCAC
TGF-β1_Human	CCCCTACATTTGGAGCCTGG	GCACGATCATGTTGGACAGC
IL6__Human	GAGGAGACTTGCCTGGTGAAA	GCAGGAACTGGATCAGGACTTT
