# Supplementary material for: Defective Ribosome Biogenesis Drives Tubular Senescence and Renal Fibrosis via IRBC–p53 Activation
Source: Cells. 2026 Oct 16;15(20):1888. doi: 10.3390/cells15201888 (PMC13702928; doi:10.3390/cells15201888)
Supplement: Supplementary file 1 [file cells-15-01888-s001.zip › cells-4537807-supplementary.pdf]

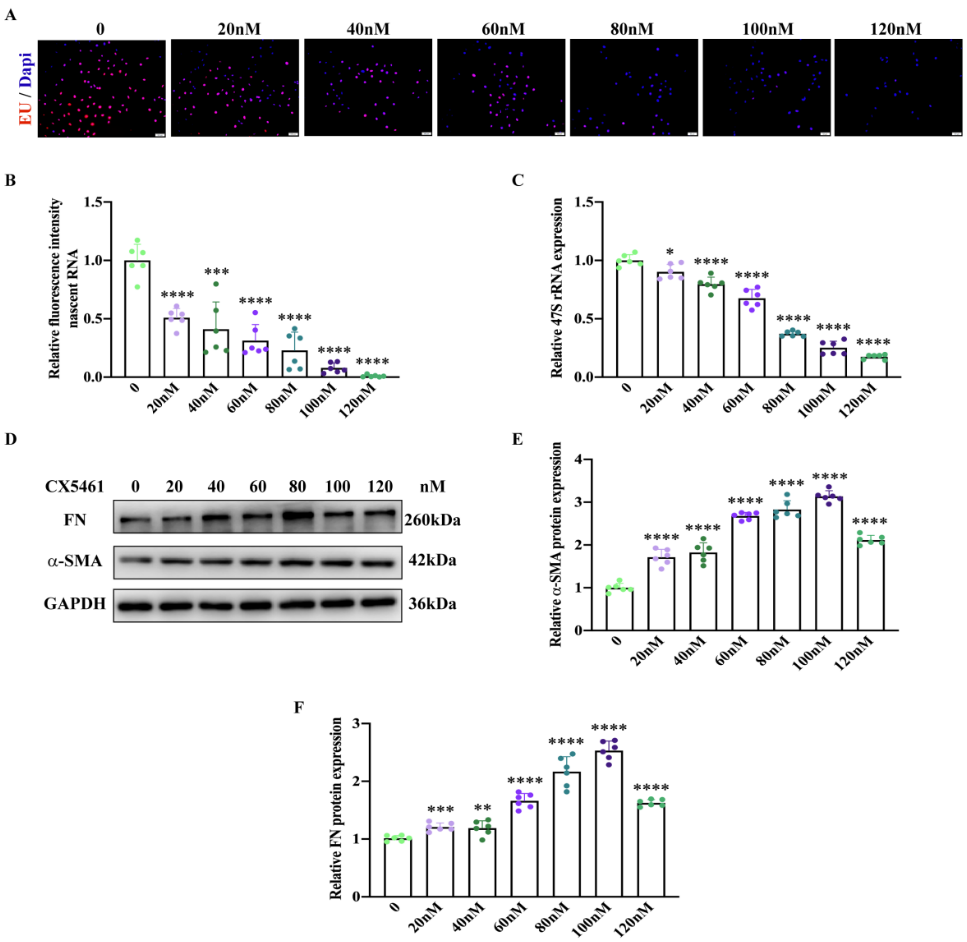

Figure S1. Inhibition of RiBi upregulated fibrotic markers in vitro. A, B Nascent RNA synthesis in control and CX5461 treated HK-2 cells was assessed by EU staining and quantified by mean fluorescence intensity analysis. C Expression level of 47S rRNA was measured by RT-qPCR. D-F Protein expression levels of α-SMA and FN were detected by western blot and semi-quantitatively analysis. n=6, one-way ANOVA followed by Bonferroni's post hoc test was used. ns, not significant, *p < 0.05, **p < 0.01, ***p < 0.001, ****p < 0.0001.

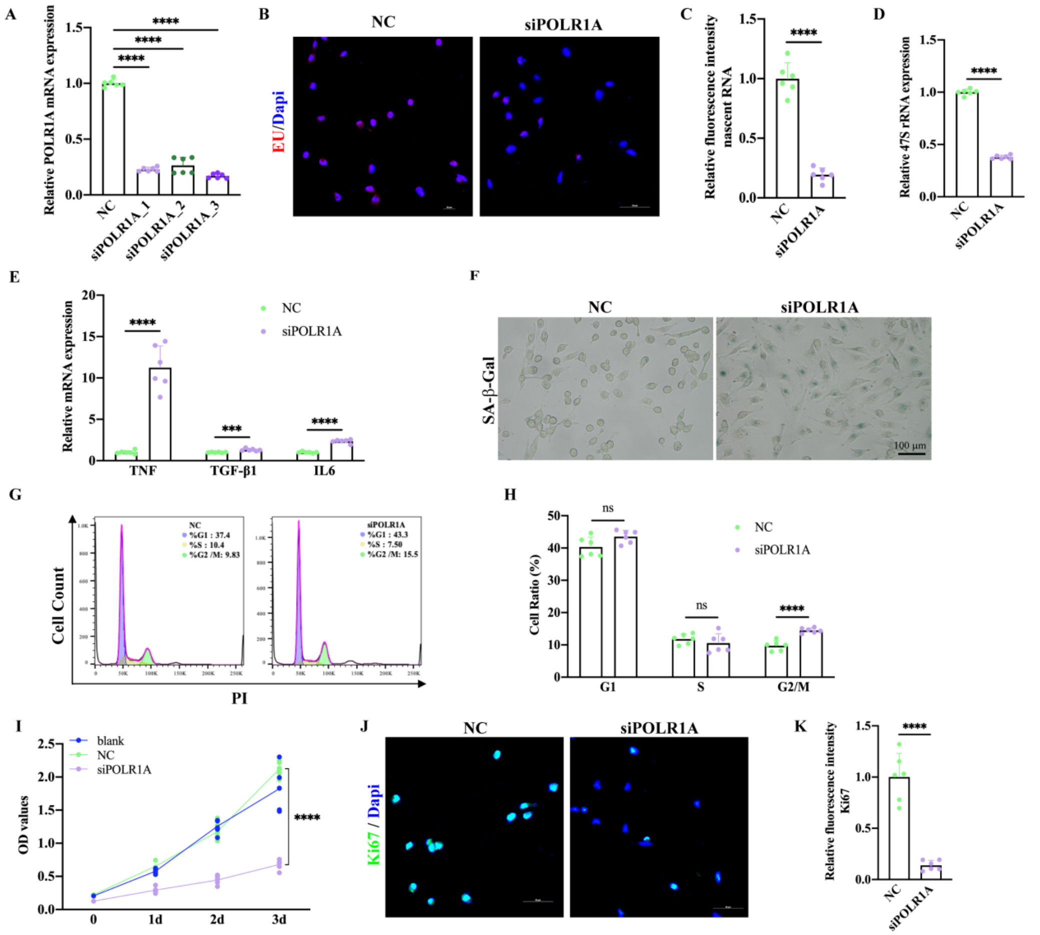

Figure S2. The impairment of RiBi caused by downregulation of POLR1A led to cellular senescence. A Expression level of POLR1A mRNA was measured by RT-qPCR. B, C Nascent RNA synthesis in NC and siPOLR1A cells was assessed by EU staining and quantified by mean fluorescence intensity analysis. D Expression level of 47S rRNA was measured by RT-qPCR. E Expression levels of TNF, TGF-β1, and IL6 mRNAs were measured by RT-qPCR. F SA-β-gal activity was measured by staining in NC and siPOLR1A treated cells. G, H Cell cycle distribution was analyzed by flow cytometry and the percentages of cells in each phase were statistically summarized. I Cell proliferation was evaluated using the CCK-8 assay. J, K Expression of Ki67 was detected by immunofluorescence and quantified by mean fluorescence intensity analysis. n=6, unpaired t-test was used in C, D, E, H, K, and one-way ANOVA followed by Bonferroni's post hoc test was used in A, I. ns, not significant, *p < 0.05, **p < 0.01, ***p < 0.001, ****p < 0.0001.

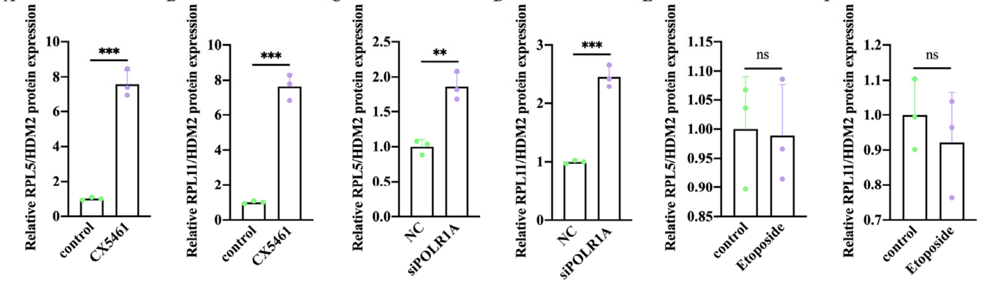

Figure S3. IRBC level analysis. A, B The IP protein expression level of RPL5/HDM2 and RPL11/HDM2 in control and CX5461 groups. C, D The IP protein expression level of RPL5/HDM2 and RPL11/HDM2 in NC and siPOLR1A groups. E, F The IP protein expression level of RPL5/HDM2 and RPL11/HDM2 in control and Etoposide groups. n=3, unpaired t-test was used. ns, not significant, *p < 0.05, **p < 0.01, ***p < 0.001, ****p < 0.0001.

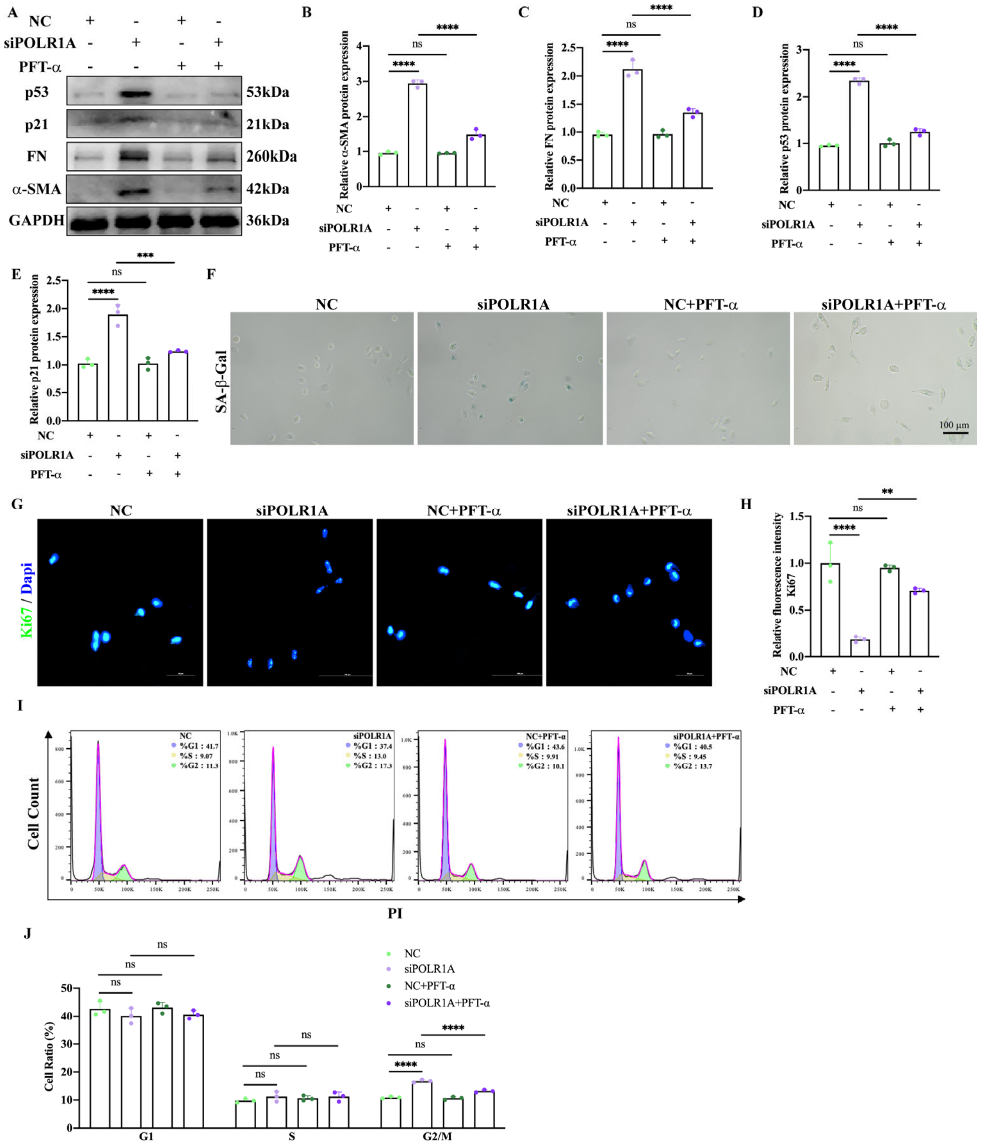

Figure S4. Inhibition of p53 alleviates cell senescence and fibrosis induced by RiBi impairment. A-E Protein expression levels of p53, p21, α-SMA and FN were detected by western blot and semi-quantitatively analysis in NC, siPOLR1A, NC+PFT-α and siPOLR1A+PFT-α groups. F SA-β-gal activity was measured by staining. G, H Expression of Ki67 was detected by immunofluorescence and quantified by mean fluorescence intensity analysis. I,J Cell cycle distribution was analyzed by flow cytometry and the percentages of cells in each phase were statistically summarized. n=3, one-way ANOVA followed by Bonferroni's post hoc test was used. ns, not significant, *p < 0.05, **p < 0.01, ***p < 0.001, ****p < 0.0001.

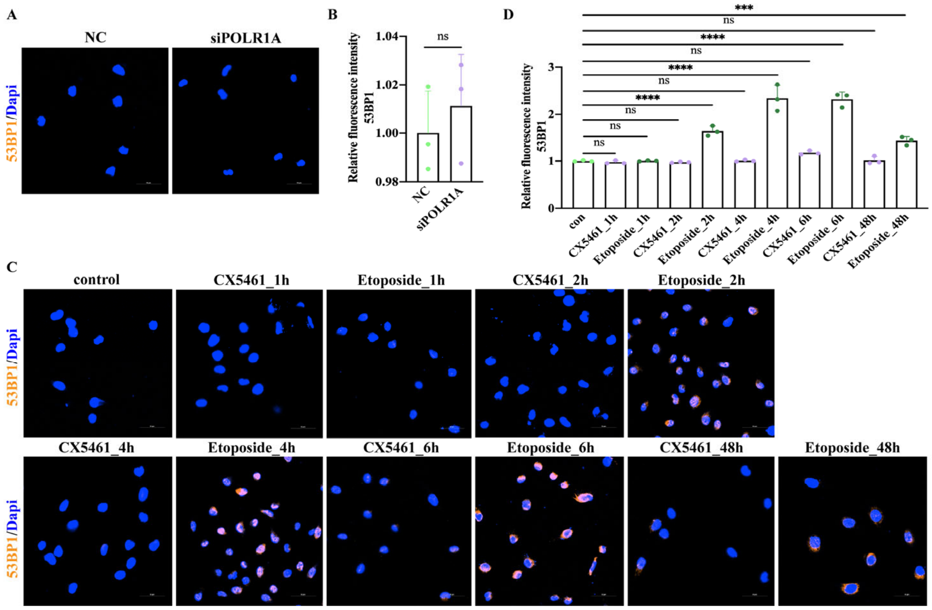

Figure S5. 53BP1 foci level in RiBi deficiency and DNA damage. A,B Expression of 53BP1 was detected by immunofluorescence and quantified by mean fluorescence intensity analysis in cells transfected with siNC or siPOLR1A. C,D Expression of 53BP1 was detected by immunofluorescence and quantified by mean fluorescence intensity analysis in HK-2 cells treated by CX5461 or Etoposide at different time points. n=3, unpaired t-test was used in B, and one-way ANOVA followed by Bonferroni's post hoc test was used in D. ns, not significant, *p < 0.05, **p < 0.01, ***p < 0.001, ****p < 0.0001.

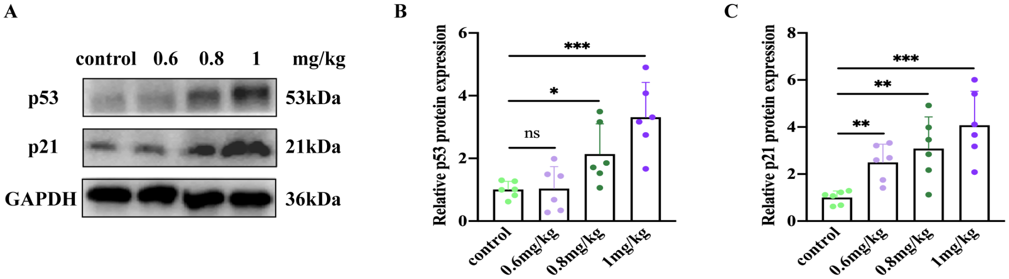

Figure S6. Senescence markers of RiBi impairment model in vivo. A-C Protein expression levels of p53 and p21 were detected by western blot and semi-quantitatively analysis in control and CX5461 treated mice. n=6, one-way ANOVA followed by Bonferroni's post hoc test was used. ns, not significant, *p < 0.05, **p < 0.01, ***p < 0.001, ****p < 0.0001.

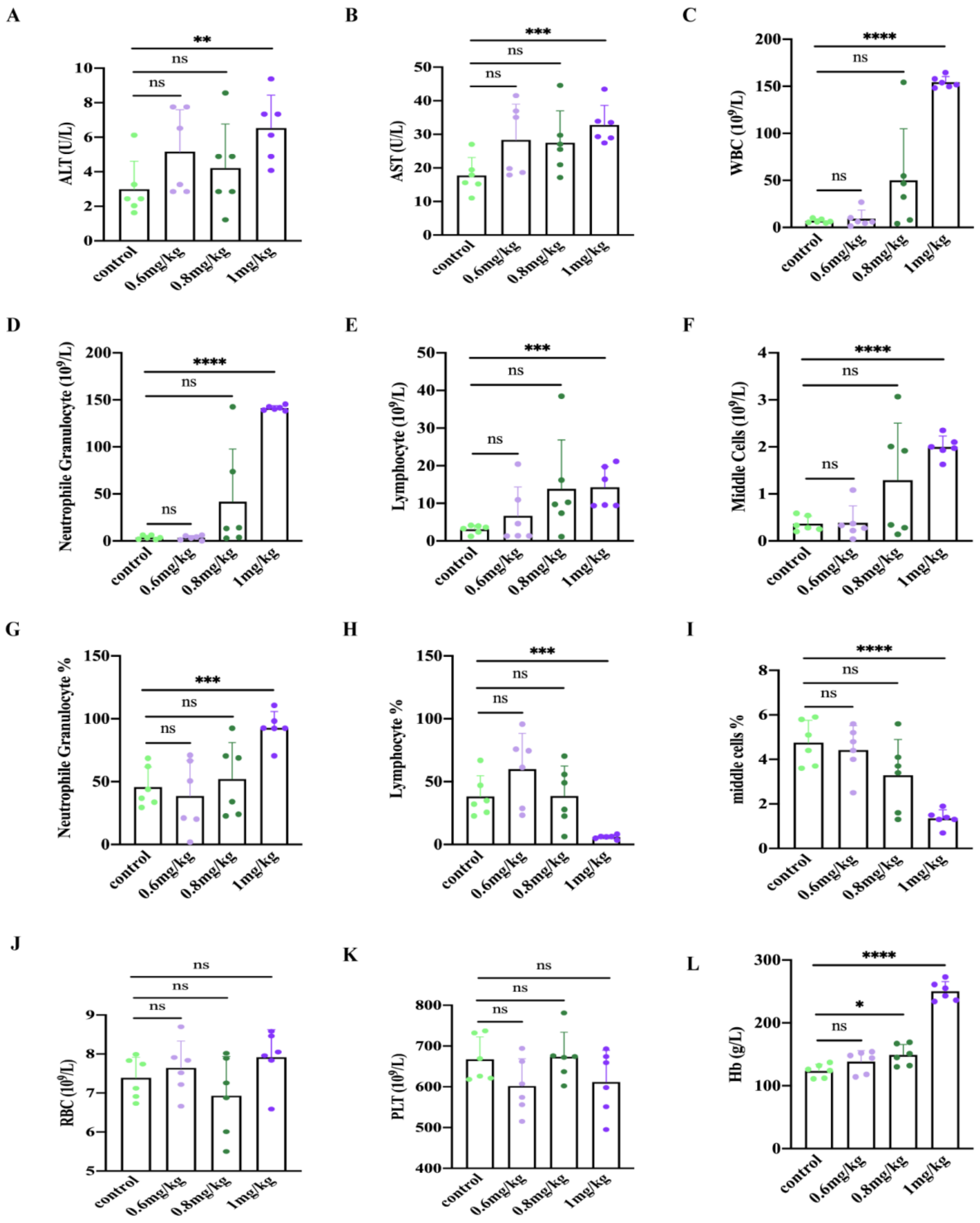

Figure S7. CX5461-induced RiBi impairment led to hepatotoxicity and altered hematological parameters in mice. A, B Assessment of liver function by measuring serum levels of alanine aminotransferase (ALT) (A) and aspartate aminotransferase (AST) (B). C-I Complete blood count analysis of immune cells. Total white blood cell (WBC) count, neutrophil count, lymphocyte count, intermediate cell count (monocytes, eosinophils, basophils), and their respective percentages are shown. J-L Analysis of erythrocyte series: red blood cell (RBC) count, platelet (PLT) count, and hemoglobin (HGB) concentration. n=6, one-way ANOVA followed by Bonferroni's post hoc test was used. ns, not significant, *$p < 0.05$, **$p < 0.01$, ***$p < 0.001$, ****$p < 0.0001$.
